# Basement membrane product, endostatin, as a link between inflammation, coagulation and vascular permeability in COVID-19 and non-COVID-19 acute respiratory distress syndrome

**DOI:** 10.3389/fimmu.2023.1188079

**Published:** 2023-05-22

**Authors:** Katharina Jandl, Johannes Lorenz Berg, Anna Birnhuber, Elisabeth Fliesser, Izabela Borek, Benjamin Seeliger, Sascha David, Julius J. Schmidt, Gregor Gorkiewicz, Martin Zacharias, Tobias Welte, Horst Olschewski, Akos Heinemann, Malgorzata Wygrecka, Grazyna Kwapiszewska

**Affiliations:** ^1^ Otto Loewi Research Center, Division of Pharmacology, Medical University of Graz, Graz, Austria; ^2^ Ludwig Boltzmann Institute for Lung Vascular Research, Graz, Austria; ^3^ Otto Loewi Research Center, Division of Physiology and Pathophysiology, Medical University of Graz, Graz, Austria; ^4^ Department of Respiratory Medicine/Infectious Diseases, Hannover Medical School, Member of the German Lung Center (DZL), Hannover, Germany; ^5^ Institute of Intensive Care, University Hospital Zurich, Zurich, Switzerland; ^6^ Department of Nephrology and Hypertension, Hannover Medical School, Hannover, Germany; ^7^ Diagnostic and Research Institute of Pathology, Medical University Graz, Graz, Austria; ^8^ Division of Pulmonology, Medical University of Graz, Graz, Austria; ^9^ Center for Infection and Genomics of the Lung, Universities of Giessen and Marburg Lung Center, Member of the German Lung Center (DZL), Giessen, Germany; ^10^ Institute for Lung Health, Member of the German Lung Center (DZL), Giessen, Germany

**Keywords:** extracellular matrix, matrikines, acute lung injury, neutrophils, endothelium, platelets

## Abstract

**Background:**

Immune cell recruitment, endothelial cell barrier disruption, and platelet activation are hallmarks of lung injuries caused by COVID-19 or other insults which can result in acute respiratory distress syndrome (ARDS). Basement membrane (BM) disruption is commonly observed in ARDS, however, the role of newly generated bioactive BM fragments is mostly unknown. Here, we investigate the role of endostatin, a fragment of the BM protein collagen XVIIIα1, on ARDS associated cellular functions such as neutrophil recruitment, endothelial cell barrier integrity, and platelet aggregation *in vitro*.

**Methods:**

In our study we analyzed endostatin in plasma and post-mortem lung specimens of patients with COVID-19 and non-COVID-19 ARDS. Functionally, we investigated the effect of endostatin on neutrophil activation and migration, platelet aggregation, and endothelial barrier function *in vitro.* Additionally, we performed correlation analysis for endostatin and other critical plasma parameters.

**Results:**

We observed increased plasma levels of endostatin in our COVID-19 and non-COVID-19 ARDS cohort. Immunohistochemical staining of ARDS lung sections depicted BM disruption, alongside immunoreactivity for endostatin in proximity to immune cells, endothelial cells, and fibrinous clots. Functionally, endostatin enhanced the activity of neutrophils, and platelets, and the thrombin-induced microvascular barrier disruption. Finally, we showed a positive correlation of endostatin with soluble disease markers VE-Cadherin, c-reactive protein (CRP), fibrinogen, and interleukin (IL)-6 in our COVID-19 cohort.

**Conclusion:**

The cumulative effects of endostatin on propagating neutrophil chemotaxis, platelet aggregation, and endothelial cell barrier disruption may suggest endostatin as a link between those cellular events in ARDS pathology.

## Introduction

Immune cell infiltration, endothelial cell barrier disruption, and widespread signs of thrombosis are key pathological features during the early phases of acute respiratory distress syndrome (ARDS) (1). Clinically, ARDS is caused by lung injury due to various insults, the most common ones being bacterial or viral infections. Amongst viral infections, ARDS caused by SARS-CoV-2 presents a subgroup among patients with severe ARDS (2).

On the vascular level, this injury activates endothelial cells leading to loss of barrier integrity. This results in an influx of protein-rich edema fluid and immune cell infiltration, followed by the rapid release of inflammatory cytokines and proteases from immune cells and structural cells. This pro-inflammatory environment further potentiates the injury, propagates immune cell infiltration, and activates a pro-thrombotic cascade (3, 4).

During these early events, epithelial and endothelial cell apoptosis denudes and exposes the underlying basement membrane (BM). The BM is a specialized extracellular matrix (ECM) compartment, which forms a sheet-like structure underneath all endothelial and epithelial cells. In the lung alveolar space, a fused BM is shared between alveolar epithelial and capillary endothelial cells (5). Exposure of the BM during injury and concomitant increased protease activity can lead to the release of BM fragments, with a novel biological function distinct from their parent molecules; these molecules are known as matrikines. Endostatin is a matrikine of collagen XVIIIα1 (encoded by COL18A1) and is currently suggested to be an early biomarker for non-COVID-19 and COVID-19 associated ARDS (6, 7). However, whether endostatin is a mere biomarker or whether it also functionally contributes to disease pathogenesis in ARDS has not been investigated so far.

In this study, we asked whether endostatin contributes to three major cellular events in early ARDS: neutrophil activation, endothelial cell barrier breakdown, and platelet activation and aggregation. In brief, we found comparable levels of endostatin in COVID-19 vs. non-COVID-19 ARDS and that it localized in proximity to the endothelium, immune cells and microthrombi in the lungs of both groups of patients. Functionally, endostatin enhanced barrier disruption, and platelet aggregation, and induced neutrophil migration. Our results point to endostatin as an important contributor to early disease propagation by functionally linking hallmark pathological features of ARDS.

## Material and methods

### Study cohort/patient’s characteristics

Lung tissue samples from critically ill COVID-19 patients and autopsy controls were collected post mortem at autopsy at the Medical University of Graz. Control patients were sex-matched and did not show signs of underlying lung pathologies. The time from death to autopsy was matched between donors and patients (8). Lung tissue from non-COVID-19 ARDS patients was collected at the Justus-Liebig-University under standard conditions as described previously (9). The study protocol and tissue usage were approved by the local ethics committees (Graz samples: EK-number: 32-362 ex 19/20 and Giessen samples: 29/01), and written informed consent was obtained for all samples derived from the Giessen cohort from all participants or their next of kin. The patient collectives and respective controls were described in detail previously (9–11). Following autopsy all tissues were fixed for 24 hours in 4% formaldehyde and embedded in paraffin for further analyses. Immunohistological slides where examined by trained pathologists.

Plasma samples were collected from 21 critically ill COVID-19 patients and 22 healthy controls at the Hanover Medical School, Hanover, Germany and from the Department of Clinical Immunology and Transfusion Medicine at the Justus-Liebig University of Giessen, Germany. Plasma samples from non-COVID-19 ARDS (all developed ARDS following influenza infection) were provided by the Hanover Medical School. Samples were collected within 6 days after the onset of ARDS. All investigations were approved by local ethics committees (Hanover samples: SEPSIS/ARDS Registry 8146_BO_K_2018, Giessen samples: ethic votum no.: 05/00). Written informed consent was obtained from all participants or their next of kin. Baseline demographics of patients and controls can be found in **Table 1** and have been previously described in detail (9, 11).

**Table 1:** Patients’ characteristics.

	COVID-19 ARDS	Non-COVID-19 ARDS	Control
**n**	21	21	22
**Age, y (SD)**	57 (+/- 15)	50 (+/- 5)	51 (+/- 14)
**Sex (m/f)**	19/2	15/7	12/6
**BMI (SD)**	33 (+/- 10)	27 (+/- 5)	NA
**CRP (SD)**	160 (+/- 64)	266 (+/- 122)	NA
**ECMO (yes/no)**	5/16	6/14	–
**Vasopressors (yes/no)**	21/0	18/3	–
**Survival (yes/no)**	18/3	16/5	–

All values depicted as mean. BMI, body mass index; CRP, C-reactive protein; ECMO, extracorporeal membrane oxygenation; NA, not available; SD, standard deviation.

### Cytokine measurements

Plasma levels of E-selectin, P-selectin, L-selection, ICAM-1, VCAM-1 and CD31 were determined using a customized LEGENDplex™ Assay (BioLegend). Sandwich High Sensitivity ELISA kits were used to quantify plasminogen activator inhibitor-1 (PAI-1), von Willebrand factor (vWF, both Thermo Fisher Scientific), endostatin, VE-cadherin, and thrombomodulin plasma levels (all from R&D). A human FXII ELISA Kit (Abnova, Taipei, Taiwan) was used to quantify levels of factor FXII. Cytokine quantification of IL-6, TNF-α, IL-8 and MCP-1/CCL2 was performed using a LEGEND MAX™ assay (Biolegend).

### Endothelial cell culture and maintenance

Human lung microvascular endothelial cells (hMVECs) were cultured as previously described (12). In brief, hMVECs were purchased from Lonza (Basel, Switzerland) and cultured in EGM MV2 with microvascular endothelial cell supplementary kit C-22121 (Lonza, Basel, Switzerland) on cell culture ware coated with 1% gelatin until they reached 90% confluency. Cells were used from passage 4 to 7.

### Endothelial cell resistance measurement

Endothelial cell resistance was recorded using the Electrical Cell-substrate Impedance Sensing System (ECIS, AppliedBiophysics, NY, USA) as previously described (13). In brief, hMVECs were seeded on ECIS 8W10E+ chips coated with 1% gelatin in full media. After reaching confluency, cells were cultured under serum-reduced conditions in EBM-2 basal medium (Lonza) supplemented with 2% fetal calf serum (FCS; Thermo Fisher Scientific) for one hour and baseline was recorded for 2 hours. Cells were pretreated with 1 µg/mL endostatin (Peprotech, Rocky Hill, NJ) for one hour followed by stimulation with thrombin (Sigma Aldrich, Missouri, USA).

### Immunohistochemistry

Formalin-fixed paraffin-embedded lung tissue sections from COVID-19 and non-COVID-19 ARDS and healthy controls were used for immunohistochemistry, and processed as previously described (13). Following heat-induced antigen retrieval in DAKO antigen retrieval solution pH6, unspecific binding sites were blocked, and slides were incubated with the following antibodies over night at 4°C: anti-Collagen IVα1 (1:100, Abcam ab6331; Cambridge, UK); anti-Endostatin (1:100, Abcam ab3453; Cambridge, UK).

### Blood collection and preparation of platelet-rich plasma and peripheral blood neutrophils

Following informed consent, blood was sampled from healthy donors (Medical University of Graz; approval no.: 17-291 ex 05/06). 3.7% sodium citrate was used as anticoagulant. Platelet rich plasma (PRP) was obtained by centrifugation at 300 g for 20 minutes. Following the removal of PRP, human peripheral blood polymorphonuclear cells were isolated by density gradient centrifugation, as previously described (14).

### Neutrophil shape change and chemotaxis

Neutrophil shape change and chemotaxis were performed as previously described (15). In brief, for shape change assays, 10,000 freshly isolated neutrophils were treated with increasing concentrations of endostatin or interleukin (IL)-8 (as a positive control) for four minutes at 37° in a water bath. Cells were immediately fixed using the BD cytofix fixation buffer (BD Life Sciences, Franklin Lakes, NJ, USA). Shape change was determined by an increase in forward scatter properties as measured on the BD Canto II (Becton Dickinson, Mountain View, CA). For chemotaxis measurements, freshly isolated neutrophils were placed on the upper compartment of a 48-well micro-Boyden chemotaxis chamber (Neuro Probe) and allowed to migrate towards endostatin for one hour at 37°C. The upper and lower compartments were separated by a PVP-free polycarbonate filter membranes with a pore size of 3 µm (Neuro Probe). Neutrophils that migrated to the lower compartment were collected and enumerated by flow cytometry (BD FACSCanto II). Flow cytometric measurement for shape change and chemotaxis were analyzed using the FlowJo v10.7.1 software (BD Life Sciences).

### Neutrophil apoptosis

Freshly isolated human peripheral blood neutrophils were incubated with endostatin (concentrations as indicated) or vehicle for 24 hours in full media (RPMI + 10% FCS) in a humidified chamber at 37°C. After 24 hours, neutrophil survival was assessed using the flow cytometric based Annexin V/Propidium Iodide Detection Kit according to the manufacturer’s instructions (BD Pharmingen, Vienna, Austria). Cells were measured on the (BD FACSCanto II) and analyzed using the FlowJo 10.7.1 software.

### Platelet activation assay

Platelet rich plasma was washed twice with Hank’s buffered saline solution without Ca^2+^ and Mg^2+^ and centrifuged for 20 minutes without breaks at 500 g. Platelets were pretreated with endostatin for 30 minutes, following 0.5-1 U/mL thrombin for two minutes, or 10 nM ADP treatment for 10 minutes at room temperature under shaking conditions. Platelets were immediately fixed using the BD cytofix fixation buffer (Fisher Scientific) and stained for CD63 (CD63 FITC clone H5C6, Biolegend, San Diego, CA, USA) and CD62P (CD62P (P-selectin) super bright 600; clone Psel.KO2.3, Biolegend, San Diego, CA, USA) according to the manufacturer’s instructions. Cell surface expression was determined by flow cytometry on the BD FACSCanto II and analyzed using the FlowJo v10.7.1 software (BD Life Sciences).

### Platelet aggregation

Platelet aggregation was performed as previously described (16). In brief, the response to a range of ADP (5-20 µM) was tested before each experiment, and concentrations that lead to 70–90% aggregation were used for platelet stimulation. Platelets were preincubated with endostatin for 20 min at 37°C. Aggregation was recorded using an Aggrecorder-II (KDK Corp, Kyoto, Japan).

### Statistical analysis

Statistical analysis were performed in R (v4.1.2) or using GraphPad Prism (v8.0, GraphPad Software, Inc., San Diego, CA, USA). Group differences for numerical values were assessed using the Mann-Whitney U test for independent samples and paired Student’s T test for dependent variables. Correlations were analyzed using the Spearman’s rank correlation coefficient. Multiple group comparisons were analyzed using one-way ANOVA followed by Dunnett’s multiple comparisons test. A detailed description of employed statistical tests is provided in the respective figure legends.

## Results

### Endostatin is elevated in COVID-19 and non-COVID-19 ARDS patients, and localizes to the endothelium, immune cells and microthrombi

**Figure 1:**
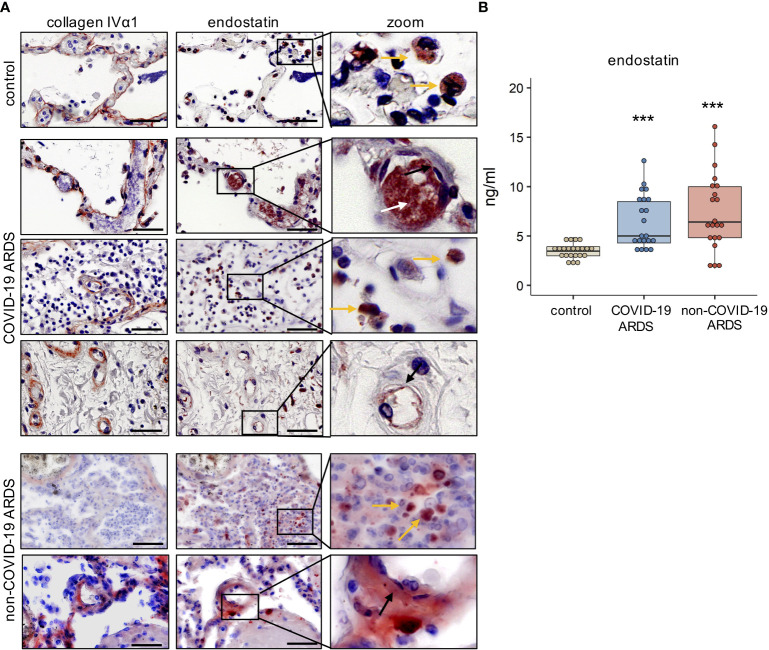
Endostatin is elevated in the lung tissue and plasma of COVID-19 and non-COVID-19 ARDS patients. **(A)** Immunohistochemistry of collagen IVα1, depicting the basement membrane, and endostatin on serial cuts of lung tissue from healthy controls, and post-mortem COVID-19 and non-COVID-19 ARDS patients. Scale bar 50 µm. Representative pictures of n=5. yellow arrow: immune cells; white arrow: microthrombi; black arrow: endothelium. **(B)** Endostatin plasma levels measured by ELISA from healthy controls, and COVID-19 and non-COVID-19 ARDS patients. ***p ≤ 0.001 compared to controls as determined by non-parametric Mann-Whitney test.

We detected enhanced immunoreactivity for endostatin in postmortem lungs of COVID-19 and non-COVID-19 ARDS patients as compared to controls. For better spatial orientation, we visualized the alveolar-capillary space by staining for the major BM protein collagen IV α1 on serial sections. In general, endostatin was found in proximity to capillary endothelial cells (black arrow), infiltrating immune cells (yellow arrow), and – if present- fibrinous clots (**Figure 1A** and **Supplementary Figure 1**). However, some interpatient – variability and slight differences in the localization pattern between COVID-19 and non-COVID-19 ARDS were observed; for example in non-COVID-ARDS, the localization to the microvasculature was more obvious, and was accompanied by a rather diffuse localization in the lung parenchyma. In our cohorts, microthrombi were only detected in COVID-19 ARDS. In the circulation, plasma concentrations of endostatin were elevated in COVID-19 and non-COVID-19 ARDS patients, at comparable levels (**Figure 1B**).

ARDS (non-COVID-19 and COVID-19) is associated with enhanced vasculopathy resulting in increased circulating levels of endothelial cell activation markers, and inflammatory and pro-thrombotic mediators. Several of these markers were previously measured in our cohorts of patients, and are reported in (9, 10).

Therefore, we next aimed to identify the role of endostatin on endothelial cell function, neutrophil recruitment and platelet activation/aggregation *in vitro*.

### Endostatin aggravates thrombin-induced endothelial cell barrier disruption, and its levels positively correlate with circulating VE-cadherin levels in COVID-19 ARDS patients

**Figure 2:**
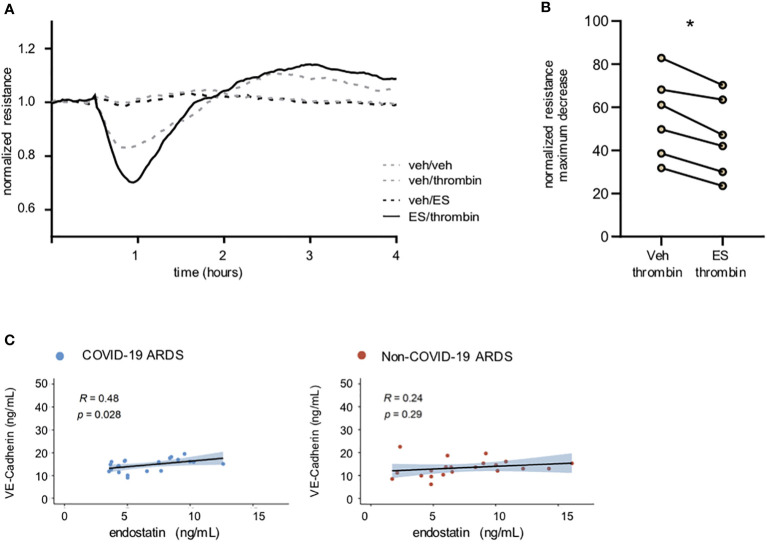
Endostatin aggravates thrombin-induced barrier disruption and correlates with soluble Ve-Cadherin in COVID-19 ARDS patients. **(A)** Representative recording of endothelial cell resistance. Pre-treatment of human microvascular EC with endostatin (1 µg/mL) enhanced the barrier disrupting effect of thrombin (endothelial resistance recorded by ECIS Z Theta; thrombin was added 30 min after establishment of a stable baseline; depicted are the statistics using paired t-test of the maximum decrease in resistance **(B)** quantification of **(A)** presented as % of stable baseline of n=6 using 3 individual hMVECs sources. * p ≤ 0.05 as determined by non-parametric Mann-Whitney test. **(C)** Spearman’s rank coefficient correlation analysis of plasma VE-Cadherin with endostatin.in non-COVID-19 and COVID-19 ARDS patients.

Loss of endothelial cell barrier integrity and vascular leakage are prominent features common to all types of ARDS. To identify the effect of endostatin on endothelial cell function, we recorded the endothelial cell barrier resistance in real time using the ECIS system. Pre-treatment of human microvascular endothelial cells (hMVECs) with 1 µg/mL endostatin aggravated the thrombin-induced barrier disruption (**Figures 2A, B**). No effect was observed with endostatin treatment alone (**Figure 2A**), or in combination with other barrier disrupting agents such as IL-4 or tumor necrosis factor (TNF)-α (data not shown). In our COVID-19 ARDS group, circulating endostatin levels showed a weak positive correlation with soluble VE-cadherin levels (**Figure 2C**), a marker indicating endothelial injury when found in the circulation (17).

### Endostatin induces moderate neutrophil activation and migration, and its levels positively correlate with plasma levels of C-reactive protein in COVID-19 ARDS patients

**Figure 3:**
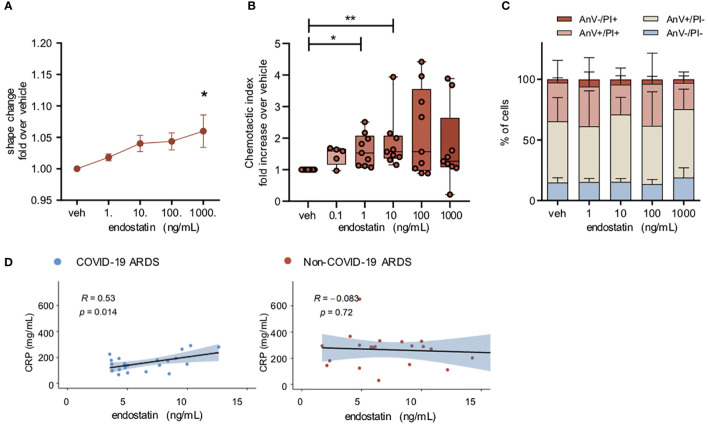
Endostatin induces a moderate neutrophil activation and migration. **(A)** Neutrophil shape change following endostatin stimulation determined by increase in forward scatter properties measured by flow cytometry. n = 4 individual healthy donors. *p ≤ 0.05 as determined by one-way ANOVA following Dunnett’s multiple comparisons test. **(B)** neutrophils were allowed to migrate to endostatin for 60 min at 37°C in a 48-well micro-Boyden chamber. Migrated cells were enumerated by flow cytometry and normalized to vehicle treated controls. n = 5 individual healthy donors. **p ≤ 0.01 as determined by one-way ANOVA following Dunnett’s multiple comparisons test. **(C)** Neutrophil apoptosis determined after 24 hours of endostatin stimulation by staining with FITC-annexin-V/propidium iodide (PI), followed by flow cytometric analysis. n = 4 individual healthy donors. **(D)** Spearman’s rank coefficient correlation analysis of plasma C-reactive protein (CRP) with endostatin in non-COVID-19 or COVID-19 ARDS patients.

Neutrophils are amongst the first cells to be recruited to the site of inflammation, and increased numbers of neutrophils in the bronchoalveolar lavage correlate with poor outcome in ARDS patients (18–21). Therefore, we next evaluated the potential role of endostatin on neutrophil function. Stimulation of isolated human peripheral blood neutrophils with endostatin led to a slight increase in neutrophil activation as determined by shape change assay (**Figure 3A**). Endostatin did not modulate the shape change induced by the neutrophil chemoattractant IL-8 (**Supplementary Figure 2**). Next, we tested, whether endostatin would also exert a chemotactic potential on human neutrophils. In line, neutrophils migrated towards endostatin, with significantly increased chemotaxis at 1 and 10 ng/mL (**Figure 3B**). Neutrophil survival after 24 hours was not affected by incubation with endostatin (**Figure 3C**). In COVID-19 ARDS patients, the proinflammatory acute phase protein, C-reactive protein (CRP) showed a weak positive correlation with endostatin while no significant correlation was observed in the non-COVID-19 ARDS cohort (**Figure 3D**).

### Endostatin enhances platelet activation and aggregation, and its levels positively correlate with circulating fibrinogen in COVID-19 ARDS patients

**Figure 4:**
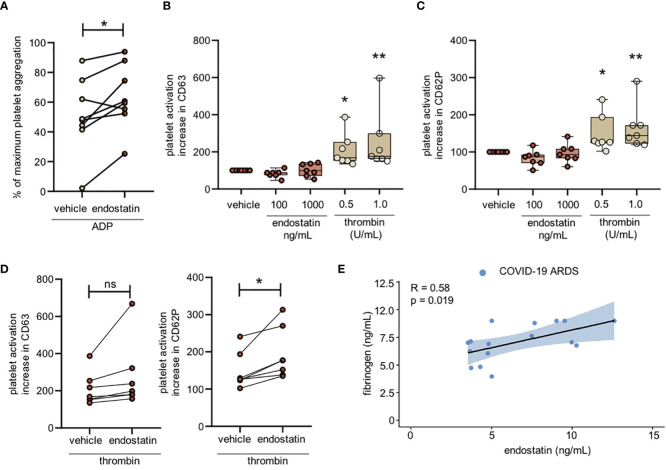
Endostatin enhances platelet activation and correlates with fibrinogen in COVID-19 ARDS patients. **(A)** Platelets were isolated from platelet-rich plasma and pretreated with endostatin (100 ng/mL) followed by stimulation with ADP. Platelet aggregation was measured using the Aggrecorder. n = 8 individual donors. *p ≤ 0.05 as determined by paired Student’s t-test. **(B)** Platelet activation determined by cell surface expression of CD63 and CD62P following endostatin treatment. n = 5-12 individual donors. Data is non-significant as determined by by one-way ANOVA following Dunnett’s multiple comparisons test. **(C)** Platelet activation determined by cell surface expression of CD63 and CD62P following thrombin treatment. n = 6 individual donors; *p ≤ 0.05 as determined by one-way ANOVA following Dunnett’s multiple comparisons test. **(D)** Platelets were pretreated with endostatin (100 ng/mL) for 20 minutes followed by stimulation with 0.5 U/mL thrombin for 2 min. Cell surface expression of CD63 and CD62P was determined by flow cytometry. n = 6 individual donors; *p ≤ 0.05 as determined by paired Student’s t-test. **(E)** Spearman’s rank coefficient correlation analysis of plasma fibrinogen with endostatin in COVID-19 ARDS patients. **p ≤ 0.01; ns, non significant.

Increased aggregation due to a prothrombotic environment is another hallmark of ARDS in general, with an even higher implication in COVID-19 ARDS (1). Therefore, we next investigated the function of endostatin on platelet activation and aggregation. We found that pre-treatment of platelets with endostatin enhanced the ADP-induced aggregation (**Figure 4A**). Detailed investigation into platelet activation revealed that endostatin treatment alone did not alter the cell surface expression of the platelet activation markers CD63 and CD62P (**Figures 4B, C**). However, pre-treatment with endostatin potentiated the thrombin-induced upregulation of CD62P on platelets, indicating a priming effect of endostatin on platelets (**Figure 4D**). A similar trend was observed for ADP-induced upregulation of the platelet activation markers, however this did not reach significance (**Supplementary Figure 3**). These findings indicate that endostatin exerts a priming effect on platelets. Additionally, a positive correlation of plasma endostatin levels with the prothrombotic molecule fibrinogen was observed in COVID-19 ARDS patients (data was not available for ARDS or control samples; **Figure 4E**).

### Plasma endostatin levels correlate positively with circulating levels of IL-6 in COVID-19 ARDS patients

**Figure 5:**
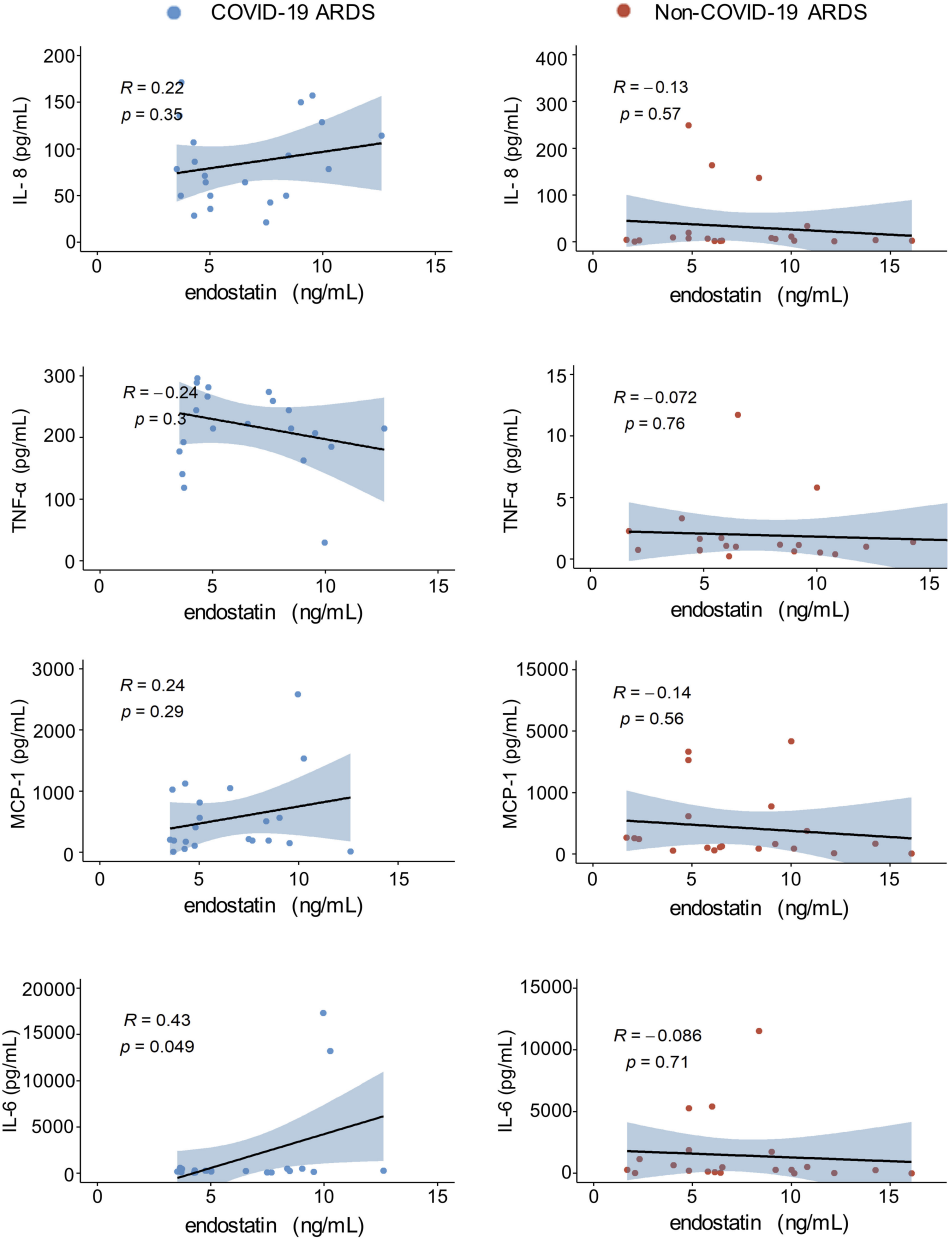
Endostatin positively correlates with IL-6 in the plasma of COVID-19 ARDS. Spearman’s rank coefficient correlation analysis of plasma IL-8, TNFα, MCP1, and IL-6 with endostatin in non-COVID-19 or COVID-19 ARDS patients. Plasma samples from COVID-19 and non-COVID-19 ARDS were analyzed in separate experiments.

A major immunological complication in COVID-19 and non-COVID-19 ARDS is the rapid release of pro-inflammatory cytokines, known as a cytokine storm (22, 23). Increased levels of cytokines such as TNF-α, IL-6, MCP-1, and IL-8 can be associated with worse clinical outcome in various forms of ARDS (22, 24, 25). In our cohorts, cytokine quantifications were performed in separate experiments, therefore, absolute values cannot be compared between the COVID-19 and non-COVID-19 group. Our correlation analysis with plasma endostatin levels revealed no correlation in the non-COVID-19 ARDS group. In COVID-19 ARDS, plasma IL-6 levels, a cytokine strongly associated with vasculopathy and endothelial cell dysfunction, positively correlated with endostatin (**Figure 5**).

Our data suggests that endostatin may have a functional role in both non-COVID-19 and COVID-19 ARDS by cumulatively acting on endothelial cells, neutrophils, and platelets.

## Discussion

Basement membrane damage and fragmentation are key pathological features observed in various types of ARDS. In line, elevated plasma levels of endostatin, the matrikine of collagen XVIIIα1, have been reported for COVID-19 and non-COVID-19 ARDS patients (6, 7). However, whether endostatin is more than a mere biomarker associated with disease severity is currently unknown. In this study, we aimed to address the question whether endostatin can actively contribute to the cellular events associated with ARDS, by focusing on neutrophil activation and migration, endothelial cell barrier integrity, and platelet activation and aggregation.

Our study reports three major findings. Firstly, we confirm elevated levels of endostatin in the plasma of patients with both COVID-19 and non-COVID-19 ARDS, which were comparable in our disease cohorts. Secondly, we demonstrate the potential of endostatin to enhance central pathological processes such as endothelial cell barrier disruption, neutrophil migration, and platelet aggregation. Thirdly, these findings are supported by several positive correlations of endostatin with circulating levels of VE-cadherin (a marker for endothelial damage), the inflammation markers CRP, and IL-6, and the coagulation factor fibrinogen in our COVID-19 cohort, while no correlations were observed in this group of non-COVID-19 patients. Although the individual correlations are only weakly positive, the cumulative effect points towards a function of endostatin in these processes. Taken together, our data presents a potential link between elevated endostatin levels and neutrophil, endothelial cell, and platelet function in early ARDS pathology.

While the angiostatic and anti-proliferative properties of endostatin on endothelial cells have been extensively studied, the impact of endostatin on barrier function has been less well characterized. Knowledge here is limited to the description of its inhibitory effect on the VEGF-induced barrier disruption in retinal microvascular endothelial cells (26). This is not surprising, as endostatin is known to inhibit all VEGF-induced actions. However, the effect of VEGF on endothelial barrier integrity is complex, with both barrier-enhancing and barrier-disrupting effects reported for concentrations of VEGF that differ only by a factor of ten (27). This suggests that blocking VEGF effects by endostatin may either protect against leakage or prevent strengthening of the barrier, depending on local VEGF concentrations. In contrast, thrombin consistently induces barrier disruption, without the ambivalent functions observed with VEGF. In this study, 1 µg/mL endostatin, a concentration that has been reported to effect endothelial cells vitro (28–30), was found to enhance thrombin-induced barrier disruption, indicating a synergistic or additive effect, although the exact mechanisms remain to be elucidated.

The loss of endothelial barrier function facilitates immune cell extravasation in ARDS (3). Neutrophils are amongst the predominant infiltrating cell type during the early stages of ARDS (4, 31, 32). Our *in-vitro* data suggested a potential of endostatin to activate and attract human neutrophils. Although the observed effects were only moderate in comparison to well-known neutrophil chemoattractants, such as IL-8 or bacterial-derived stimuli such as fMLP (N-Formylmethionyl-leucyl-phenylalanine), our findings suggest an enhancing effect of endostatin on the inflammatory environment during ARDS (33). Indeed, chemotactic potentials have been reported for other proteolytic fragments of the BM, such as PGP (which can also be released from collagen IV) or Laminin 332 peptides (34, 35).

Endothelial dysfunction and exacerbated neutrophil activation (for example *via* formation of neutrophil extracellular traps) has been linked to the pro-coagulatory environment in COVID-19 and non-COVID-19 ARDS (36–39) and altered platelet function has been reported for COVID-19 ARDS patients (9, 40, 41). Here, we observed increased activation of platelets following pretreatment with endostatin, which resulted in elevated expression of CD62P, and increased aggregation. Although further functional assays such as clotting assays, degranulation, and PAF (platelet activating factor) release would be needed to fully elucidate the role of endostatin in coagulation, this data provides first insights into a possible contribution.

Despite the comparatively weaker individual effects of endostatin on neutrophil activation, platelet aggregation, and endothelial barrier disruption, its cumulative action on all three factors could lead to a significant contribution to the pathogenesis of ARDS.

To further clarify this hypothesis, we performed multiple correlations with markers of endothelial vasculopathy, thrombosis, and inflammation; this was possible as both the COVID-19 and non-COVID-19 cohort have been well described in our previous studies (9–11). Indeed, plasma of this cohort of COVID-19 patients revealed pro-thrombotic characteristics, visible by fibrinolysis-resistant clot formation, and increased formation of fibrin-dense clots compared to non-COVID-19 ARDS (9). Similarly, circulating levels of endothelial markers such as ICAM-1, VCAM-1, and prothrombotic markers such as vWF or P-selectin have been reported in this cohort of COVID-19 ARDS patients (10). When performing correlation analyses on our COVID-19 and non-COVID-19 ARDS cohort, we observed no correlations with endostatin in the non-COVID-19 ARDS group. In the COVID-19 ARDS group, however, mild but significant positive correlations of endostatin with VE-cadherin, CRP, IL-6, and fibrinogen were detected. Furthermore, these positive correlations corroborate our *in-vitro* findings and suggest a possible cumulative role of endostatin on endothelial cell dysfunction, coagulation, and neutrophil activation in (COVID-19) ARDS.

Interestingly, similar correlations of endostatin with the inflammation marker CRP have been observed in other inflammatory pathologies with vascular involvement, such as COPD. This may suggest a more universal link between systemic inflammation and endostatin generation (42). Functional studies have suggested that CRP can deteriorate vascular injury by opsonization of endothelial cells and promotion of immune cell invasion. Whether this might be linked to endostatin release and function would need further investigation (43, 44). IL-6 is another important early phase mediator that has been associated with negative prognosis in COVID-19 patients and was suggested as a possible therapeutic target (45). Vascular injury stimulates the production of IL-6 from endothelial cells, thereby potentially contributing to the inflammatory milieu in ARDS that leads to BM fragmentation and endostatin release (46). However, the extent to which cytokine profiles and vascular inflammation differ between COVID-19-associated ARDS and non-COVID-19 ARDS is still a matter of debate (47, 48). Indeed, a large meta-analysis of cytokine measurements in ARDS patients of different causes revealed lower levels of inflammatory markers, such as IL-6 and TNF-α, in COVID-19-associated ARDS (49). In light of disease heterogeneity, larger cohort studies with specific focus on the underlying mechanisms are needed to unravel the link between endostatin release and its dependence on a specific inflammatory environment.

In general, the connection between inflammatory cytokines and endostatin appears to be multifactorial. A study in a mouse model of acute sepsis demonstrated that subcutaneous administration of endostatin improved survival and lung damage by reducing cytokine levels, including TNF-α (50). Another study demonstrated the potential of endostatin to block TNF-α-mediated NF-kB activation *in vitro*, although at extremely high concentrations of more than 12.5 µg/ml (51). Whether the pro- or anti-inflammatory effects of endostatin are dominant in ARDS would require more detailed experimental studies in disease-specific models which mimic the human inflammatory environment.

Taken together, our data show an involvement of endostatin in key pathologic processes of ARDS by regulating neutrophil function, coagulation, and endothelial barrier integrity. These modulatory effects subsequently promote a pro-inflammatory feedback loop of endothelial dysfunction, enhanced immune cell activation leading to further BM degradation and endostatin proteolysis. Although more research efforts will be necessary to assess the therapeutic potential of targeting endostatin cleavage and effects, this study contributes to understanding the complex mechanism leading to the severe characteristics of ARDS.

## Data availability statement

The raw data supporting the conclusions of this article will be made available by the authors, without undue reservation.

## Ethics statement

The studies involving human participants were reviewed and approved by EK-number: 32-362 ex 19/20; Giessen samples: 29/01; Hanover samples: SEPSIS/ARDS Registry 8146_BO_K_2018, Giessen samples: ethic votum no.: 05/00; Medical University of Graz; approval no.: 17-291 ex 05/06). The patients/participants provided their written informed consent to participate in this study.

## Author contributions

Conceptualization, KJ, GK, JLB, and MW; Data curation, KJ, JLB, MW, EF, AB, and IB; Resources, GK, AH, BS, SD, JJS, GG, MZ, TW, HO, and MW; Supervision, GK and KJ; Visualization, KJ, JLB, and AB; Writing – original draft, KJ, JLB, GK, and MW; writing – review and editing, all authors. All authors contributed to the article and approved the submitted version.
