# Supplementary material for: Basement membrane product, endostatin, as a link between inflammation, coagulation and vascular permeability in COVID-19 and non-COVID-19 acute respiratory distress syndrome
Source: Front Immunol. 2023 May 22;14:1188079. doi: 10.3389/fimmu.2023.1188079 (PMC10241244; doi:10.3389/fimmu.2023.1188079)
Supplement: Supplementary file 1 [file DataSheet_1.docx]

Supplementary Figure 1


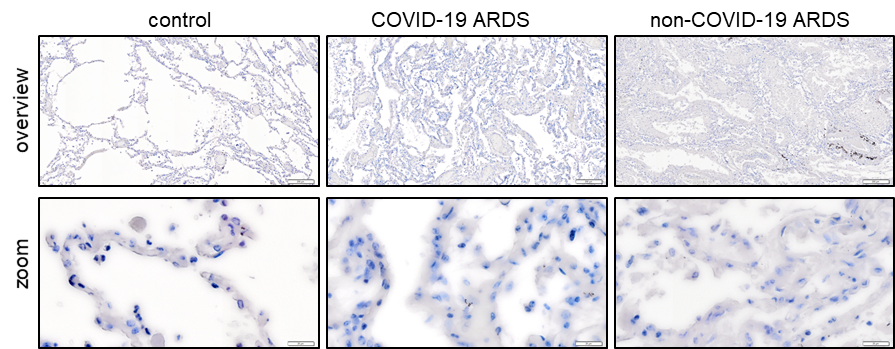


Supplementary Figure 1. Negative control stainings on control, COVID-19 ARDS, and non-COVID-19 ARDS lung tissue. Samples were treated as in Figure 1 A, with ommission of the primary antibody.

Supplementary Figure 2


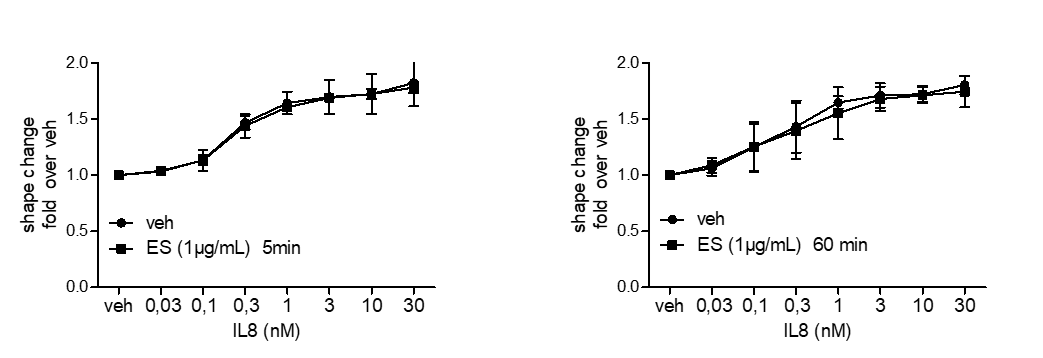


Supplementary Figure 2. Endostatin does not increase neutrophil shape change induced by IL-8. Neutrophils were pretreated with endostatin for 5 or 60 min, followed by stimulation with IL-8 for four minutes. Shape change was determined by an increase in forward scatter properties measured by flow cytometry. n = 4 individual healthy donors.

Supplementary Figure 3.


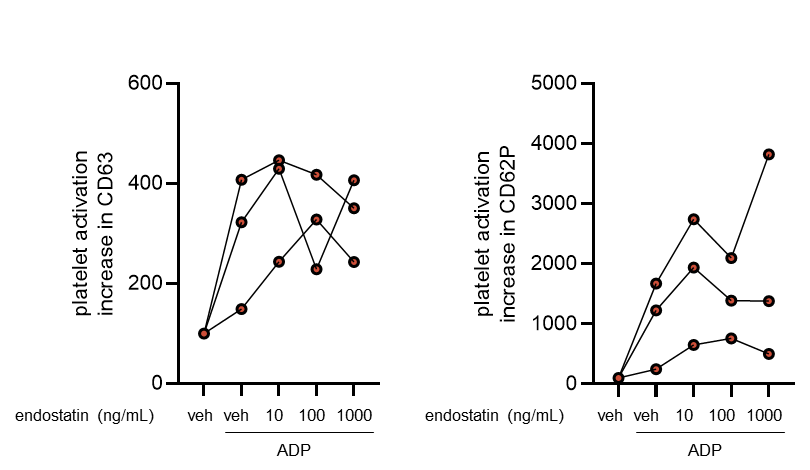


Supplementary Figure 3. Platelet activation determined by cell surface expression of CD63 and CD62P following endostatin and ADP treatment. n = 3 individual donors. Data is non significant as determined by by one-way ANOVA following Dunnett‘s multiple comparisons test.
